# Deciphering the fungal symphony: unveiling the fungal dynamics during the fermentation of traditional Chinese strong-flavor *Daqu*

**DOI:** 10.3389/fmicb.2025.1540118

**Published:** 2025-01-24

**Authors:** Lina Zhao, Wenjing Zhang, Yuting Niu, Xiaohan Chen, Jiuyang Guo, Ying Wu, Xuan Li, Shaobin Gu

**Affiliations:** ^1^College of Food and Bioengineering, Henan University of Science and Technology, Luoyang, China; ^2^Henan Engineering Research Center of Food Microbiology, Luoyang, China; ^3^National Demonstration Center for Experimental Food Processing and Safety Education, Luoyang, China

**Keywords:** *Daqu* fermentation, fungal community, interaction relationship, keystone species, MT-*Daqu*

## Abstract

*Daqu*, a starter culture rich in microorganisms like bacteria and fungi, is central to vinification and liquor brewing, yet fungal contributions are often understudied. In this study, we used Illumina MiSeq sequencing to investigate the succession of fungal community during Chinese strong-flavor liquor fermentation. The results showed that the alpha and beta diversity of fungal community were significantly different during *Daqu* fermentation. The dominant phyla and genera are Ascomycota and *Saccharomycopsis*, respectively. Co-occurrence network analysis identified 10 keystone species during *Daqu* fermentation, displaying that the positive correlations (99.64%) dominated the fungal network. The redundancy analysis showed that moisture has the most significant influence on the *Daqu* fungal community. Concurrently, a robust association was observed between 10 keystone fungal genera and environmental parameters such as acidity and moisture. These findings not only elucidated the intricate dynamics of the fungal community succession and the interplays among fungi but also pinpointed the primary drivers of the fungal community and its keystone species during the *Daqu* fermentation process. Ultimately, this research presented novel perspectives for enhancing the quality and precision of liquor production by shedding light on the central role of keystone species in maintaining community stability and their adaptive responses to environmental stimuli.

## 1 Introduction

Chinese strong-flavor liquor, a distinctive category of Chinese spirits, contained over 1,300 types of flavor compounds and was characterized by its harmonious taste and cellar aroma. Highly favored by Chinese consumers, the production and sales of Chinese strong-flavor liquor constituted approximately 70% of the total output of China’s liquor industry (He et al., 2020). *Daqu* is a kind of starter culture used in liquor making, which was mainly used for solid state fermented liquor. The *Daqu* fermentation process generated a diverse array of aromatic compounds, which endowed the liquor with its distinctive aroma and flavor profile (Wang et al., 2017a). *Daqu* is usually classified into three types, depending on the maximum temperature reached during the fermentation process, i.e., low temperature *Daqu* (LT-*Daqu*) (45–50°C), medium temperature *Daqu* (MT-*Daqu*) (50–60°C), and high temperature *Daqu* (HT-*Daqu*) (60–65°C) (Wang et al., 2011). Meanwhile, MT-*Daqu*, the most widely used starter in the production of traditional Chinese strong-flavor liquor, is rich in bacteria, fungi and enzymatic systems and serves as both the foundation of Chinese strong-flavor liquor production and the key determinant of its quality (Fan et al., 2007; Zhang et al., 2009).

During the *Daqu* production process, sophisticated techniques of natural inoculation and open fermentation were employed. These methods adeptly harnessed the microbial diversity of the brewing environment, primarily bacteria and fungi (Du et al., 2019), thereby imparting *Daqu* with its unique flavor and superior quality. Bacteria served as the crucial microbial group in the fermentation process of *Daqu* that produced protease and flavoring substances (Wang et al., 2020). In recent years, the rapid advancement of high-throughput sequencing technology had led to its widespread application in analyzing the microbial community structures across diverse environments (Bruno et al., 2016; Fan et al., 2020; Wang et al., 2017b). The Chao index (representing richness), Shannon and Simpson index (representing diversity), and coverage index (representing coverage) were used to describe the microbial composition of *Daqu* (Zhuansun et al., 2022). It has been reported that Firmicutes and Proteobacteria were the dominant bacterial phyla in the fermentation process of *Daqu* (He et al., 2019; Hu et al., 2017; Sun et al., 2016; Tao et al., 2014; Wang et al., 2017b). The dominant bacterial genera were mainly *Lactobacillus* and *Bacillus* (Ge et al., 2024; Tang et al., 2023). In addition, research on bacterial community has primarily focused on the interactions among bacterial communities and the correlations between bacterial communities and physicochemical properties, etc. (Qian et al., 2021; Zhang et al., 2020; Zhi et al., 2016). These studies not only enhanced our understanding of *Daqu’s* microbial ecology but also offered a scientific foundation for optimizing the *Daqu* fermentation process. However, studies on the succession characteristics and mechanisms of *Daqu* fungal community are still limited.

Fungal communities play a crucial role in the fermentation process of *Daqu* (Zheng et al., 2012), such as producing alcohol, enzymes, and flavor compounds (Wang et al., 2017b), and providing the necessary saccharification, liquefaction, and proteolytic capabilities required by *Daqu*. Moreover, they also produce a range of organic acids and other important substances, generating various flavor components, and enhancing the quality of *Daqu* (Wang et al., 2017c). Currently, research on the fungal communities in *Daqu* fermentation paralleled that of bacterial communities, focusing primarily on the shifts in fungal communities composition, the interactions between bacterial and fungal communities, the impact of the fungal communities on physicochemical properties, and the influence of physicochemical factors on the fungal communities (Li et al., 2019a; Safatov et al., 2010; Zhang et al., 2007). Much of the published literature relies on Redundancy analysis (RDA) to elucidate correlations between microbial community structure and physicochemical factors (Jin et al., 2019; Zhu et al., 2022a), however, the identification of keystone species in fungal communities and the keystone drivers influencing them remain unclear. Consequently, the identification of keystone fungal species may elucidate the assembly mechanisms of the *Daqu* microbial community, thereby offering technical support for enhancing *Daqu* quality and for research into its functional roles and enhancement strategies. Moreover, the response and adaptability of the keystone fungal species to environmental physicochemical factors may also have been a critical determinant in the *Daqu* fermentation process.

Therefore, this study analyzed the dynamic changes in fungal community composition at various time points throughout the MT-*Daqu* fermentation process (0, 10, 20, 30, and 120 days). The study aimed to explore (i) changes in diversity and composition of fungal community during *Daqu* fermentation, (ii) interaction and keystone species composition of *Daqu* fungal community, and (iii) key drivers of keystone species of *Daqu* fungal community.

## 2 Materials and methods

### 2.1 Sample collection

**Figure 1:**
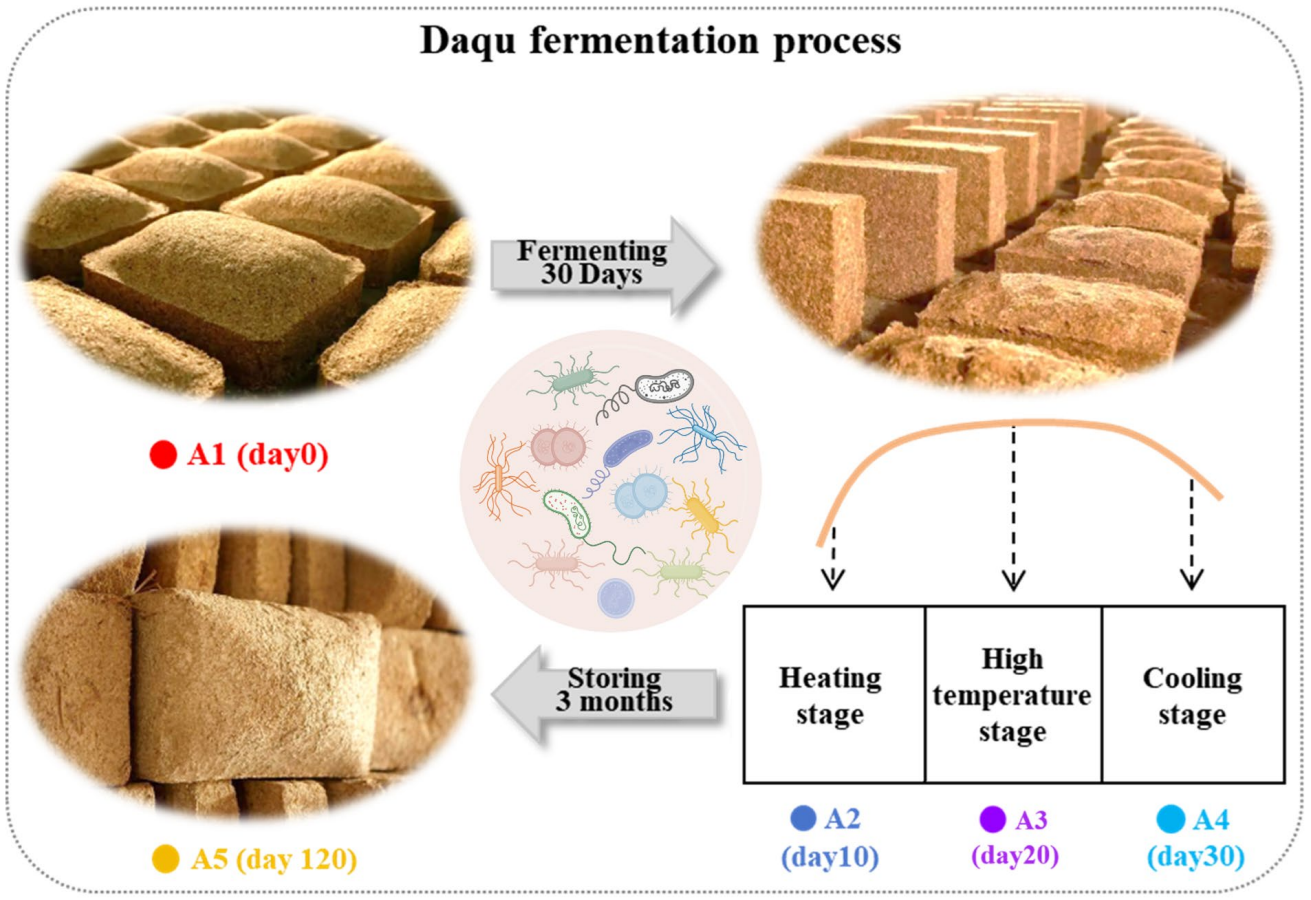
(i) Wheat was meticulously ground into *Daqu* powder and subsequently fashioned into *Daqu* bricks. Sample A1 was extracted on day 0. (ii) A natural solid-state fermentation process lasting approximately 30 days was meticulously carried out. During this period, samples A2, A3, and A4 were extracted at three distinct stages, namely the heating stage, the high temperature stage, and the cooling stage. (iii) The fermented bricks were allowed to mature in a sealed fermentation chamber for 3 months during the maturation period to facilitate further biochemical transformation. After this maturation period, sample A5 was extracted.

The *Daqu* production process consists of three key stages (Figure 1). In June 2020, MT-*Daqu* samples were collected from a renowned Chinese strong-flavor liquor distillery in Henan Province, China. Approximately 100 g of *Daqu* were sampled from the upper, middle, and lower locations in the Qu room at different fermentation stages: 0 (A1), 10 (A2), 20 (A3), 30 (A4), and 120 days (A5) (Wang et al., 2017b). On each sampling day, the samples were immediately mixed to form a composite *Daqu* sample. Triplicate composite samples for each fermentation stage were collected using this method. Finally, all 15 samples were promptly placed in an icebox and stored at −80°C for subsequent analysis (Soergel et al., 2012).

### 2.2 DNA extraction, amplification, and sequencing

Genomic DNA was extracted from *Daqu* samples using the EZNA™ (Easy Nucleic Acid Isolation) stool DNA Kit (Omega Bio-tek, Norcross, GA) according to the manufacturer’s protocol. The corresponding primer pairs ITS1F and ITS2R (Adams et al., 2013) were used for PCR amplification of fungal ITS gene. The purified amplicons were sequenced using Illumina MiSeq platform (Allwegene Technology Co., Ltd., Beijing, China). All sequence data were submitted to the NCBI Sequence Read Archive (SRA) under accession number PRJNA1118944.

### 2.3 Sequence data processing and fungal community analysis

The raw FASTQ files were multiplexed and filtered using the QIIME1 (version 1.9.1) pipeline with the following criteria: (i) The 300-bp reads were truncated at any site receiving an average quality score < 20 over a 50-bp sliding window, discarding the truncated reads shorter than 50 bp; (ii) exact barcode matching, two nucleotide mismatch in primer matching, reads containing ambiguous characters were removed, and (iii) only sequences that overlapped >10 bp were assembled according to their overlap sequence. Subsequently, the sequences were clustered into operational taxonomic units (OTUs) at 97% sequence identity using UPARSE.^1^ Alpha diversity analysis was used to evaluate the richness of fungal communities. The overall differences in fungal community composition among *Daqu* samples were evaluated by principal co-ordinate analysis (PCoA) and permutational multivariate analysis of variance (PERMANOVA) performed with R^2^ based on the Bray–Curtis distance. “Hmisc” R package was used to construct the co-occurrence network based on the spearman correlation matrix. Correlations with Spearman’s |*ρ*| > 0.6 and *p* < 0.05 were visualized as co-occurrence network using “Gephi (version 0.9.2)” (Bastian et al., 2009), and the “hub node” was defined according to node with a high degree (> 25) and closeness centrality (> 0.4) value in co-occurrence network (Zhao et al., 2022).

### 2.4 Statistical analysis

One-way analysis of variance coupled with Tukey’s Honestly Significant Difference test was conducted using SPSS 20.0 software (SPSS Inc., Chicago, IL, USA) to ascertain the statistical differences in the Chao indices of fungal communities within *Daqu* samples. RDA was used to assess the relationship between fungal composition of *Daqu* and physicochemical properties (data from Mao et al., 2022; Supplementary Table 1). The statistical significance of the RDA was assessed by the Monte Carlo Permutation test with 499 permutations, and the analytical program CANOCO 5.0 was used (Ter Braak and Šmilauer, 2012). Heatmap was generated using OmicShare tools,^3^ and other figures were generated using Origin 8.0 (Origin Lab Corporation, Northampton, MA, USA).

## 3 Results

### 3.1 Diversity of *Daqu* fungal community

**Figure 2:**
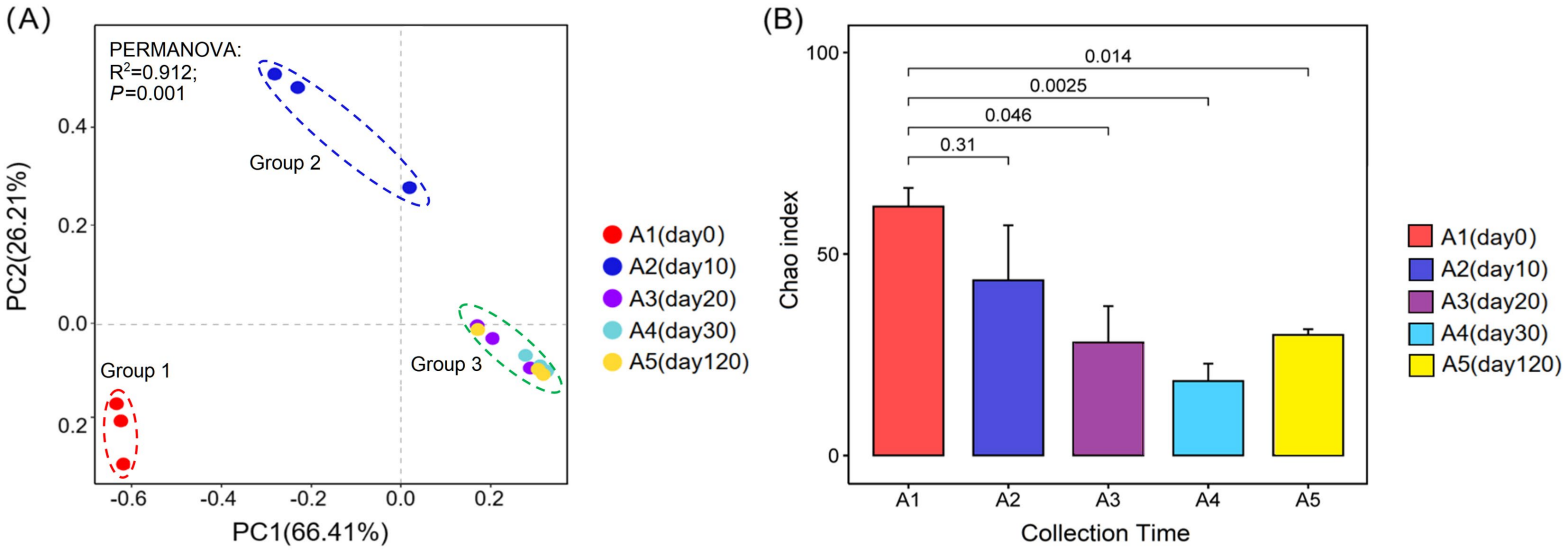
Diversity of fungal community in *Daqu* samples at the OTU level (97% similarity). **(A)** PCoA of fungal community based on Bray–Curtis distances. **(B)** Chao index of fungal community. A1-A5, fermented grains that fermented for 0, 10, 20, 30, or 120 days.

The analysis of *β*-diversity using PCoA revealed that the first principal component (PC1) explained 66.41% of the variation, and the second principal component (PC2) accounted for an additional 26.21% (Figure 2A). Together, these principal coordinates provided a combined interpretation degree of 92.62%. There were significant differences in the composition of *Daqu* fungal community in the early stages of fermentation (day 0 and day 10). However, the composition of the fungal community in *Daqu* was similar during the late fermentation stage (20, 30, and 120 days) (Figure 2A). Additionally, the richness of the fungal community in *Daqu* first decreased and then increased during the fermentation process (Figure 2B).

### 3.2 Taxonomic composition of *Daqu* fungal community

**Figure 3:**
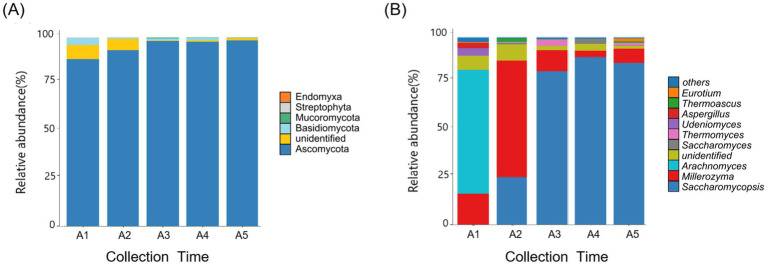
**(A)** The relative abundance of *Daqu* fungi at the phylum level. **(B)** The relative abundance of *Daqu* fungi at the genus level. Others, non-abundant taxa in the fungal community. Data are expressed as the mean of three replications. A1-A5, fermented *Daqu* that fermented for 0, 10, 20, 30, or 120 days.

At the phylum level, Ascomycota was detected as abundant phyla in *Daqu* fungal samples. The relative abundance of ascomycetes stabilized at 97% after 20 days of *Daqu* fermentation (Figure 3A). At the genus level, based on the criterion of maximum relative abundance, *Arachnomyces* was identified as the dominant genus at the onset of the fermentation process (day 0). Subsequently, the relative abundance of *Millerozyma* was dominant (day10). Concurrently, *Saccharomycopsis* began to emerge on day 10, gradually increasing in abundance and eventually becoming the most dominant strain within the sample. By the 30th day of fermentation, *Saccharomycopsis* reached its peak, constituting 90% of the total fungal communities (Figure 3B).

### 3.3 Network analysis of *Daqu* fungi community

**Figure 4:**
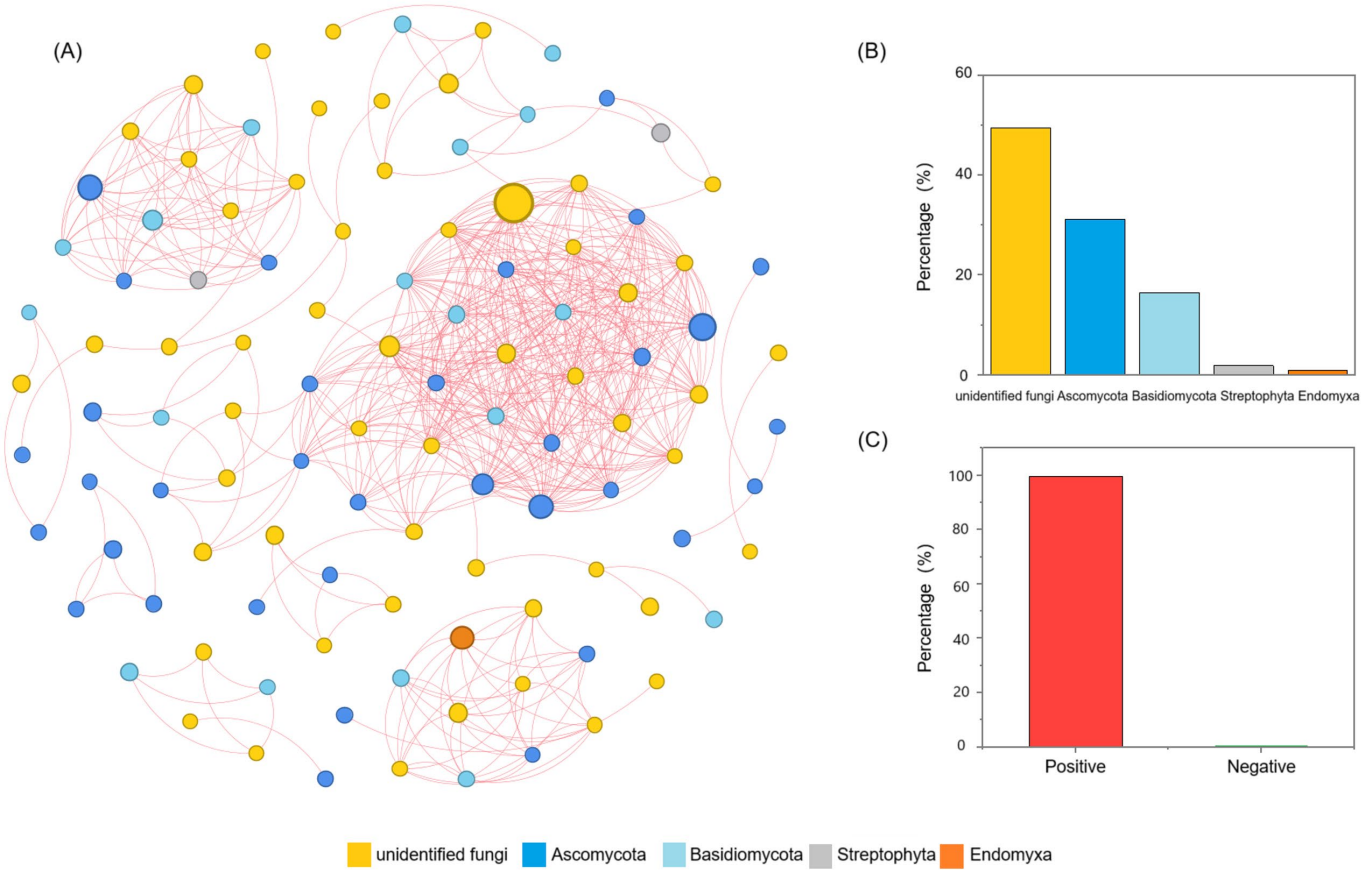
Networks analysis of the relationships between the Daqu fungal community. **(A)** Co-occurrence network of Daqu fungi based on correlation analysis. A connection stands for a strong (Spearman’s |*ρ*| > 0.6) and significant (*p* < 0.05) correlation. The size of each node is proportional to the number of connections. **(B)** Proportion of unidentified-fungi, Ascomycota, Basidiomycota, Streptophyta and Endomyxa in the network. **(C)** Proportion of positive and negative connections in the network.

In order to explore the interactions among fungal communities during the fermentation process of *Daqu*, network analysis based on Spearman’s rank correlations (|*ρ*| > 0.6 and *p* < 0.05) were constructed and visualized (Figure 4A). The network contained 109 nodes, the unidentified fungi accounted for 49.54%, Ascomycota 31.19%, Basidiomycota 16.51%, Streptophyta 1.83%, and Endomyxa 0.92% (Figure 4B). Meanwhile, 560 pairs of positive correlations (99.64%) and 2 pairs of negative correlations (0.36%) were identified from fungal communities (Figure 4C). In addition, based on the values of degree (> 25) and closeness centrality (> 0.4), the co-occurrence network had 10 hub nodes, which were considered as keystone taxa. These keystone taxa were the keystone species of the *Daqu* fungal community, including OTU 117 (*Udeniomyces pyricola*), OTU 146 (*unidentified fungi*), OTU 151 (*Aspergillus penicillioides*), OTU 152 (*Alternaria* sp.), OTU 22 (*Udeniomyces megalosporus*), OTU 25 (*Udeniomyces megalosporus*), OTU 53 (*unidentified fungi*), OTU 63 (*unidentified fungi*), OTU 67 (*Arachnomyces jinanicus*), and OTU 70 (*Arachnomyces jinanicus*).

### 3.4 Key drivers of *Daqu* fungal community

**Figure 5:**
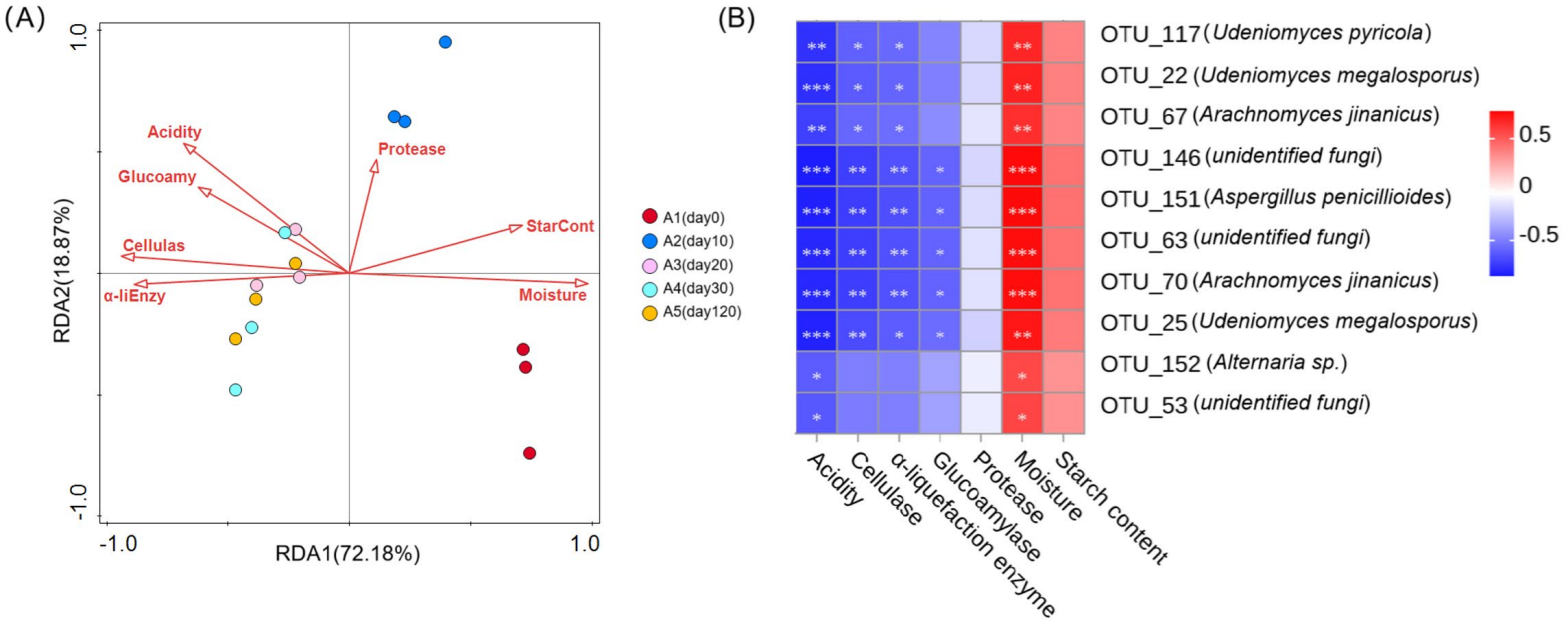
**(A)** RDA of physicochemical factors in relation to fungal compositional structures. The arrow represents the direction and magnitude of the physicochemical factors associated with the fungal community structure, and the length of the arrow denotes the intensity of the correlation. A1-A5, fermented grains that fermented for 0, 10, 20, 30 or 120 days. **(B)** Heat map of relevance between 10 keystone species and physicochemical factors. The legend on the right shows the color interval of different R values; (* 0.01 < *p* ≤ 0.05, ** 0.001 < *p* ≤ 0.01, *** *p* ≤ 0.001).

The RDA showed that the explanation rates of the fungal community structure differences were 72.18% and 18.87%, respectively (Figure 5A). Significant exploratory variables for the differences in the fungal community structure during *Daqu* fermentation was moisture (70%; *F* = 30.4; *p* = 0.002) (Figure 5; Supplementary Table 2). Concurrently, moisture exhibited a negative correlation with acidity, saccharification enzyme activity, cellulase activity, and *α*-amylase activity. To further elucidate the factors driving the keystone species within the *Daqu* ecosystem, we correlated the 10 dominant fungal species with various physicochemical factors. The analysis showed that moisture had a significant positive correlation with the 10 keystone fungi genera (*p* < 0.05). Conversely, acidity showed a significant negative correlation with these genera (*p* < 0.05). In addition, significant positive associations with cellulase and α-amylase activity were found in OTU 117 (*Udeniomyces pyricola*), OTU 22 (*Udeniomyces megalosorus*), and OTU 67 (*Arachnomyces jinanicus*). In the other hand, OTU 146 (*unidentified fungi*), OTU 151 (*Aspergillus penicillioides*), OTU 63 (*unidentified fungi*), OTU 70 (*Arachnomyces jinanicus*) and OTU 25 (*Udeniomyces megalosorus*) exhibited a significant negative correlation with cellulase, α-amylase, and glycosylase enzyme activities (*p* < 0.05). At the same time, there was no significant correlation between protease and starch levels and 10 keystone fungi genera (Figure 5B).

## 4 Discussion

Understanding the succession patterns and interaction dynamics of the *Daqu* fungal community is crucial for the production of Chinese strong-flavor liquor. Although the composition and functionality of numerous *Daqu* fungal community have been examined, research into the keystone species and their specific roles has been somewhat constrained. Therefore, a systematic analysis of the role and function of the keystone species within the *Daqu* fungal community is essential for optimizing the brewing process of Chinese strong-flavor liquor.

Our initial study findings predominantly disclosed a pronounced disparity in fungal diversity between the initial sampling on day 10 and subsequent samplings. Notably, the fungal communities during the pre-fermentation stage (day 0) were significantly different from those observed during the fermentation phase (from day 0 to day 30) and the storage phase (from day 30 to day 120). Ascomycota emerged as the predominant fungal phylum in the fermentation process of *Daqu*, aligning with previous research findings (Dong et al., 2024; Tong et al., 2023; Zhou et al., 2023). Additionally, a pronounced shift in the microbial community structure was observed between days 10 and 20 of the fermentation process. The relative abundance of *Saccharomycopsis* increased progressively throughout the fermentation process, ultimately becoming the predominant strain within the *Daqu* fungal community. *Saccharomycopsis*, a prevalent microbial flora in *Daqu*, is capable of producing *α*-amylase and glucoamylase, thereby significantly enhancing the glycosylation capacity of the mixture (Chen et al., 2021; Li et al., 2019a; Xie et al., 2021).

A notable secondary finding of our study was that the synergistic interactions among keystone taxa within the *Daqu* fungal community are crucial for maintaining its stability. Network hubs, identified as potential keystone taxa, play a pivotal role in the stability of community structure and function (Banerjee et al., 2019; Delgado-Baquerizo et al., 2016; Xiong et al., 2020). Employing co-occurrence network analysis in this study, we identified keystone species within the fungal fermentation process. Unidentified fungi, along with *Udeniomyces pyricola*, *Aspergillus penicillioides*, *Udeniomyces megalosorus*, *Arachnomyces jinanicus* and *Alternaria* sp., were revealed as keystone taxa that play a critical role in the dynamics of the fungal community. Although specific contributions of *Udeniomyces pyricola* and *Udeniomyces megalosorus* to the *Daqu* fermentation process are not explicitly detailed in the literature, their classification within the yeast genus suggests potential synergistic interactions with yeasts in analogous environments, potentially contributing to liquor fermentation and the development of distinctive flavors. It is plausible that these species may have influenced the fermentation process and flavor profile. Future research may provide more detailed insights into the role of these yeasts in *Daqu*. Concurrently, the specific role of *Arachnomyces jinanicu*s warrants further elucidation. We hypothesize that *Arachnomyces jinanicus* may secrete a range of enzymes involved in the metabolic pathways within the *Daqu* fermentation environment, potentially influencing the synthesis of flavor compounds and thus impacting the taste and aroma profiles of the liquor product. *Aspergillus penicillioides*, capable of secreting a diverse array of hydrolytic enzymes including *α*-amylase, glycosylase, glucosidase, endoglucanase, and *β*-glucosidase, promotes the succession of the *Daqu* microbial community and the fermentation of fermented grains (Li et al., 2019b; Xia et al., 2022). *Alternaria* sp., a keystone functional fungal genus within the wheat microbiota, plays a significant role in various metabolic pathways during the early stages of incubation. Finally, the unidentified fungi may also have held a unique role in the ecosystem, and additional research might have been warranted to explore and ascertain their specific ecological and economic significance. In conclusion, keystone species within the *Daqu* fermentation process likely influenced the assembly, succession, stability, and adaptability of the entire microbial community through robust biological interactions. These interactions ultimately shaped the flavor and aroma profiles of Chinese strong-flavor liquor.

Moreover, given the paramount importance of physicochemical factors in the fermentation process of liquor, precise management and modulation of *Daqu* fungal community were essential during the initial phase of fermentation (Azzolini et al., 2012; Lu et al., 2015; He et al., 2022). As the microbial succession progressed, the role of deterministic selection became increasingly significant (Dini-Andreote et al., 2015), and the keystone species within the *Daqu* ecosystem under specific conditions were found to exhibit a correlation with their environmental determinants (Mao et al., 2022; Wang et al., 2021). During the fermentation process, the keystone microbial strains were primarily influenced by the levels of moisture and acidity. Moisture, acting as a vital energy substrate for the microbial community, was essential for catalyzing a range of biochemical reactions within the *Daqu* matrix (Ma et al., 2021). These processes included starch hydrolysis, protein degradation and esterification processes, which were fundamental to the biosynthesis of the key flavor compounds that imparted the distinctive characteristics to the distilled spirit. In addition, the acidity exerted a significant impact on the growth and metabolic activities of microorganisms within the fermentation medium. An optimal acidic environment was not only instrumental in fostering the proliferation and reproduction of beneficial microorganisms, including bacteria and yeast, which contributed to the establishment of a dominant microbiota, but it also served to suppress the proliferation of detrimental microorganisms. The meticulous regulation of the acid generation range throughout the fermentation process was crucial for ensuring the smooth progression of Chinese strong-flavor liquor fermentation (Zhu et al., 2022b).

## 5 Conclusion

This study conducted a comprehensive analysis of the composition and diversity within the *Daqu* fungal community, with a focus on identifying keystone species and their pivotal roles. The research revealed that moisture and acidity were the primary environmental determinants influencing these keystone species. The findings systematically elucidated the succession patterns and underlying mechanisms of fungal communities during the *Daqu* fermentation process, enhancing our understanding of the keystone species’ critical function in maintaining community stability and their adaptive responses to environmental changes. Consequently, this study offered novel insights into the intricate dynamics of fungal interactions in the production of Chinese strong-flavor liquor and served as a valuable reference for the future optimization and selection of *Daqu* fungal community.

However, there were several limitations that might need to be addressed in our future work. First, the use of amplicon-based sequencing, as opposed to metagenomics, constrains the study’s capacity to fully capture the functional potential of the fungal community. Second, a larger sample size is needed to enhance statistical power and reduce the likelihood of false positives. In addition, in order to better understand the driving effects of keystone taxa and physicochemical factors on fungal community succession during *Daqu* fermentation, it is necessary to expand the sampling range of *Daqu* samples and carry out a comprehensive tracking and monitoring project on fungal community and *Daqu* ecosystem characteristics.

## Data Availability

The datasets presented in this study can be found in online repositories. The names of the repository/repositories and accession number(s) can be found at: https://www.ncbi.nlm.nih.gov/, PRJNA1118944.

## Footnotes

^1^ https://drive5.com/uparse/

^2^ http://www.r-project.org/

^3^ https://www.omicshare.com/tools/
