# Supplementary material for: Deciphering the fungal symphony: unveiling the fungal dynamics during the fermentation of traditional Chinese strong-flavor Daqu
Source: Front Microbiol. 2025 Jan 24;16:1540118. doi: 10.3389/fmicb.2025.1540118 (PMC11802508; doi:10.3389/fmicb.2025.1540118)
Supplement: Supplementary file 1 [file Table_1.DOC]

**Table 1 Determination results of physicochemical indexes and hydrolase systems during the fermentation period of strong-flavor *Daqu.***

| **Fermentation**  **time (days)** | **Moisture**  **（%）** | **Acidity**  **(mmol/10g)** | **Starch content (g/100g)** | **Cellulase**  **(U/mg)** | **Protease**  **(U/g)** | **Glucoamylase**  **(U/mg)** | **a-liquefaction enzyme (U/mL)** |
| --- | --- | --- | --- | --- | --- | --- | --- |
| Day 0 | 30.12±0.01 | 0.66±0.16 | 52.31±0.38 | 0.29±0.05 | 2333.90±360.22 | 144.45±26.26 | 0.13±0.00 |
| Day 10 | 20.26±0.03 | 1.63±0.12 | 51.47±2.00 | 0.47±0.05 | 3571.57±213.65 | 215.38±14.48 | 0.23±0.00 |
| Day 20 | 10.90±0.00 | 1.55±0.09 | 35.57±3.98 | 0.56±0.06 | 3693.57±141.57 | 205.84±6.55 | 0.29±0.01 |
| Day 30 | 8.14±0.01 | 1.27±0.11 | 49.50±2.57 | 0.72±0.01 | 3019.92±130.94 | 212.77±30.67 | 0.41±0.00 |
| Day 120 | 6.56±0.00 | 1.91±0.16 | 30.62±0.77 | 0.65±0.02 | 788.57±84.59 | 283.88±40.33 | 0.49±0.00 |

**Table 2** Explanation of physicochemical factors on variation of *Daqu* fungal community structure.

| **Name** | **Explains %** | ***F*** | ***P*** |
| --- | --- | --- | --- |
| Moisture (%) | 70 | 30.4 | 0.002 |
| Acidity (mmol/10g) | 10.14 | 6.4 | 0.012 |
| Protease (U/g) | 5.3 | 4.1 | 0.03 |
| Glucoamylase (U/mg) | 3.3 | 3 | 0.072 |
| a-liquefaction enzyme (U/mL) | 1 | 0.9 | 0.442 |
| Cellulase (U/mg) | 0.7 | 0.6 | 0.536 |
| Starch content (g/100g) | 0.9 | 0.7 | 0.45 |
